# Damage evaluation in graphene underlying atomic layer deposition dielectrics

**DOI:** 10.1038/srep13523

**Published:** 2015-08-27

**Authors:** Xiaohui Tang, Nicolas Reckinger, Olivier Poncelet, Pierre Louette, Ferran Ureña, Hosni Idrissi, Stuart Turner, Damien Cabosart, Jean-François Colomer, Jean-Pierre Raskin, Benoit Hackens, Laurent A. Francis

**Affiliations:** 1 ICTEAM Institute, Université catholique de Louvain, Place du Levant 3, 1348 Louvain-la-Neuve, Belgium; 2 Research Group on Carbon Nanostructures (CARBONNAGe), University of Namur, Rue de Bruxelles 61, 5000 Namur, Belgium; 3 Department of Physics, Research Center in Physics of Matter and Radiation (PMR), University of Namur, Rue de Bruxelles 61, 5000 Namur, Belgium; 4 NAPS/IMCN, Université catholique de Louvain, 2 Chemin du Cyclotron, 1348 Louvain-la- Neuve, Belgium; 5 Electron Microscopy for Materials Science (EMAT), Department of Physics, University of Antwerp, Groenenborgerlaan 171, B-2020 Antwerp, Belgium; 6 Institute of Mechanics, Materials and Civil Engineering, Université catholique de Louvain, Place Sainte Barbe 2, B-1348 Louvain-la-Neuve, Belgium

## Abstract

Based on micro-Raman spectroscopy (μRS) and X-ray photoelectron spectroscopy (XPS), we study the structural damage incurred in monolayer (1L) and few-layer (FL) graphene subjected to atomic-layer deposition of HfO_2_ and Al_2_O_3_ upon different oxygen plasma power levels. We evaluate the damage level and the influence of the HfO_2_ thickness on graphene. The results indicate that in the case of Al_2_O_3_/graphene, whether 1L or FL graphene is strongly damaged under our process conditions. For the case of HfO_2_/graphene, μRS analysis clearly shows that FL graphene is less disordered than 1L graphene. In addition, the damage levels in FL graphene decrease with the number of layers. Moreover, the FL graphene damage is inversely proportional to the thickness of HfO_2_ film. Particularly, the bottom layer of twisted bilayer (t-2L) has the salient features of 1L graphene. Therefore, FL graphene allows for controlling/limiting the degree of defect during the PE-ALD HfO_2_ of dielectrics and could be a good starting material for building field effect transistors, sensors, touch screens and solar cells. Besides, the formation of Hf-C bonds may favor growing high-quality and uniform-coverage dielectric. HfO_2_ could be a suitable high-K gate dielectric with a scaling capability down to sub-5-nm for graphene-based transistors.

One of the most explored application domains for graphene is nanoelectronics because of its high carrier mobility and atomic thickness^1^,^2^. However, gate dielectric deposition is an important challenge for transferring graphene transistors from laboratory level to industrial production. Dielectric or metal deposition induces defects in monolayer (1L) graphene and at the interface between dielectric and few-layer (FL) graphene^3^. The carrier mobility is very sensitive to the graphene lattice defects and interface quality. It has indeed been reported that the carrier mobility of suspended graphene is significantly higher than that of graphene lying over a silicon dioxide (SiO_2_) substrate^4^ due to corrugation, traps at the interface and fixed charges in the dielectric layer. Therefore, it is a crucial task to grow metal-oxide dielectric films on graphene with minimum damage/degradation into the graphene lattice.

Although physical vapor deposition (such as magnetron sputtering), widely used in the semiconductor industry, can provide high deposition rates and preserve film stoichiometry, it generates extensive damage in graphene from high energy sputtered atoms^5^. Researchers mostly choose atomic layer deposition (ALD)^6^,^7^ for dielectric growth on graphene^8^. ALD allows for controlling the thickness and uniformity of the deposited films with atomic-level precision while avoiding physical damage of energized atoms to the surface. ALD techniques are classified into plasma (oxygen-based) and thermal (water-based) depositions. Very few examples dealing with the former technique were reported. Only Nayfeh *et al.*^9^ demonstrated a graphene transistor for which aluminum oxide (Al_2_O_3_) gate dielectric was directly deposited on graphene by using a remote plasma-enhanced ALD (PE-ALD) process. Most of the reports are related to the latter method, because plasma is rather aggressive (especially using a direct plasma) and generally etches graphene^10^. It is well known that graphene is hydrophobic and inert. More specifically, graphene does not provide reactive nucleation sites for the precursors in thermal ALD^11^,^12^ since it does not display covalent bonds out of the plane. Therefore, growing high-quality and uniform-coverage dielectrics by thermal ALD requires a graphene pretreatment. Various approaches have been proposed: (i) graphene is chemically modified by fluorine^13^, ozone^14^, nitride plasma^15^,^16^, organic molecules^17^ or perylene tetracarboxylic acid^18^,^19^; (ii) metal particles are deposited on graphene as appropriate nucleation layers^20^; (iii) self-assembled monolayers are used to template the direct growth of dielectrics^21^; (iv) graphene islands, serving as a seed layer, are generated by low-power plasma^22^. Some of these approaches are complicated and incompatible with the existing mainstream integrated circuit technology. More particularly, these approaches might cause undesirable side effects, such as: inducing defects, doping graphene, leaving seed layers, increasing the dielectric thickness, and degrading the dielectric properties. Two previous articles pointed out that graphene is possibly degraded by the pretreatment^23^,^24^. Alternatively, the ozone pretreatment has proved to be responsible for significant damage to graphene in high-temperature thermal ALD^25^.

In this work, we use mild plasma conditions to directly grow hafnium oxide (HfO_2_) and Al_2_O_3_ dielectrics on graphene by PE-ALD. In our process, the graphene samples are placed away from the plasma source for outside of the glow discharge. The remote oxygen plasma with low ion bombardment avoids fast etching of graphene. Simultaneously, the reaction between graphene and the precursor still guarantees physical/chemical modification of graphene. Based on micro-Raman spectroscopy (μRS) and X-ray photoelectron spectroscopy (XPS), we study the structural damage induced in 1L graphene underlying HfO_2_ and Al_2_O_3_ upon different oxygen plasma power levels. We evaluate the damage levels in AB-stacked bilayer (AB-2L), twisted bilayer (t-BL) and trilayer (3L) graphene (hereafter they are collectively referred to as FL) underlying HfO_2_ and Al_2_O_3_ for a fixed oxygen plasma power. We also investigate the influence of the HfO_2_ thickness on graphene with various layer numbers. The results indicate that in the case of Al_2_O_3_/graphene, both 1L and FL graphene are strongly damaged under the present process conditions. In the case of HfO_2_/graphene, μRS analysis clearly shows that FL graphene presents much less disorder than 1L graphene. Moreover, the FL graphene damage decreases with the number of layers. Our results also reveal that an inverse dependence of FL graphene damage with increasing the thickness of HfO_2_ film. FL graphene allows for controlling/limiting the defect formation during the PE-ALD HfO_2_ process. Therefore, it could be a good starting material for applications such as graphene-based transistors and sensing devices^26^,^27^, since, presently, wafer-scale homogeneous FL graphene can been synthesized by chemical vapor deposition (CVD)^28^.

## Results

### Graphene morphology observations by SEM, AFM, optical microscopy and TEM

**Figure 1:**
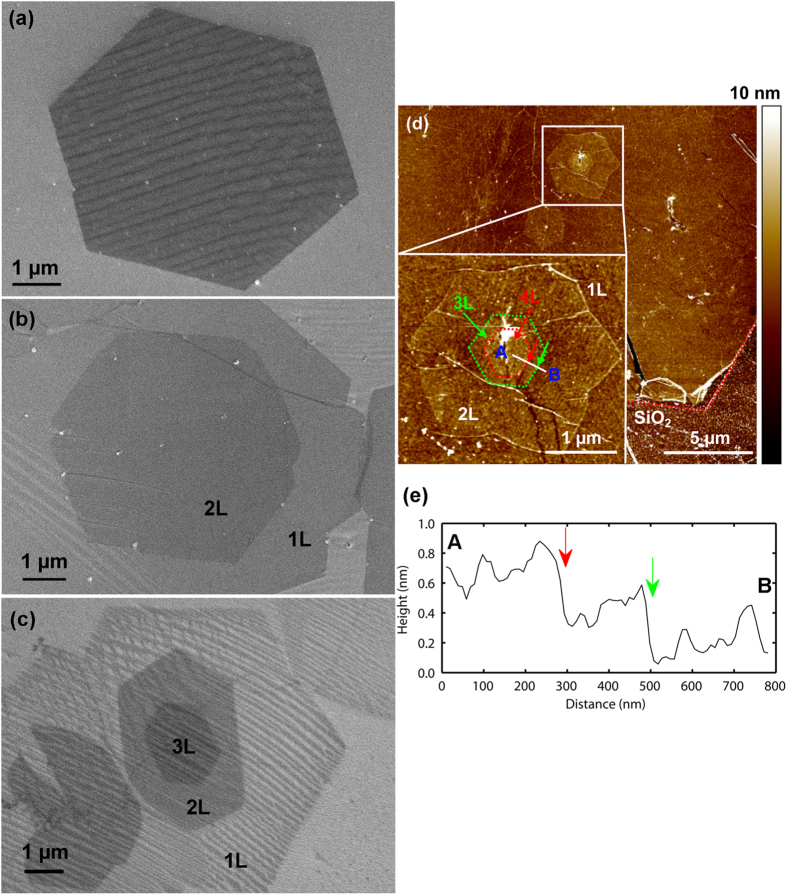
Scanning electron microscopy images for (a) monolayer layer (1L), (b) bilayer (2L), and (c) trilayer (3L) graphene on copper foils. (**d**) Atomic force microscopy image of as-transferred graphene on SiO_2_/Si substrate. (**e**) Profile of stacked graphene flakes along the A–B line.

Figure 1a–c show scanning electron microscopy images of atmospheric pressure chemical vapor deposition (APCVD) graphene on copper foils (see methods and the reference^29^ for more information about the graphene growth and transfer conditions). The different contrasts in the images correspond to the different graphene layer numbers. It can be seen that the 1L, 2L, and 3L graphene domains are hexagonal. An hexagon is the typical shape of graphene domains grown by APCVD^30^. In another article, the same group explored the shape variations *versus* the APCVD growth conditions. They explained the shape modulation by the competition between atom diffusion along graphene domain edges or corners and surface diffusion processes^31^. In addition, Fig. 1d presents an atomic force microscopy image of graphene transferred onto SiO_2_, in which 1L, 2L and 3L can be clearly seen. Figure 1e plots the profile of stacked graphene flakes along the A–B line, confirming that each graphene layer has a thickness of about 0.3 nm.

**Figure 2:**
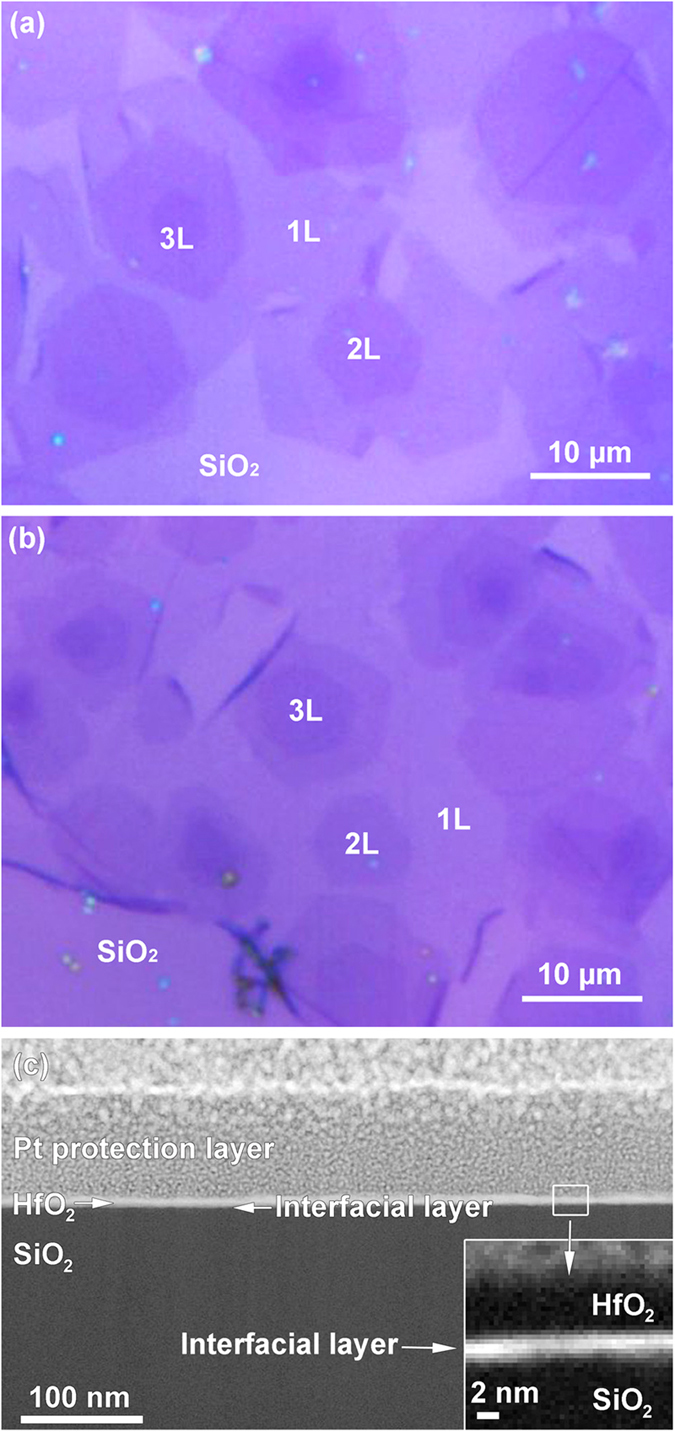
Optical and transmission HAADF-STEM images: (**a**) as-transferred graphene on SiO_2_/Si substrate, (**b**) HfO_2_/graphene/SiO_2_/Si stack and (**c**) cross-section of HfO_2_/graphene/SiO_2_/Si stack.

Figure 2a illustrates an optical microscopy image of as-transferred graphene on the SiO_2_/Si substrate, revealing that the graphene film is composed of isolated and contiguous hexagonal flakes of various layer numbers. Most of the hexagons are 1L. High-angle t-2L, AB-2L and 3L graphene hexagons are found in certain regions. As shown in Fig. 2b, graphene underlying the HfO_2_ dielectric film is still clearly visible. Figure 2c shows a cross-sectional high angle annular dark field scanning transmission electron microscopy (HAADF-STEM/Z-contrast) image of the HfO_2_/graphene/SiO_2_/Si stack, indicating the uniform covering of graphene with the HfO_2_ film. No clustering or pinholes in the HfO_2_ film is observed.

### Graphene structural damage evaluation by μRS

**Figure 3:**
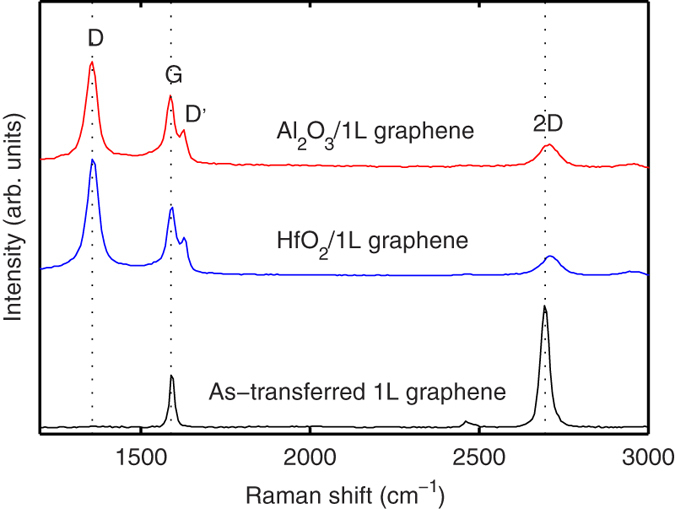
Raman spectra of monolayer graphene without/with PE-ALD HfO_2_ and PE-ALD Al_3_O_2_ films for an oxygen plasma power of 300 W.

μRS is a nondestructive method and is employed here to assess the structural damage in graphene underlying dielectric films. It is worth noting that a laser wave length of 514 nm was used in all the measurements except for the case by special description. Figure 3 shows the Raman spectra of 1L graphene after PE-ALD Al_2_O_3_ and HfO_2_, at an oxygen plasma power of 300 W. The spectra are offset for clarity. For the sake of comparison, the Raman spectrum of as-transferred 1L graphene is also shown at the bottom of the figure. According to the statistical data from many measured points, the peak at ~1588 cm^−1^ originates from the G mode of graphene. The non-perturbed G mode is usually around 1580 cm^−1^ (see the work of Ferrari *et al.*^32^), which is a first order Raman peak related to the E_2g_ optical phonons at the Brillouin zone center. The slight upshift is most probably due to residual strain originating from the copper substrate or/and unintentional doping. The doping possibly comes from traps in the SiO_2_ substrate, from insufficient rinsing after copper etching, from PMMA residues in transfer step, from moisture in air, and other similar contamination sources^33^,^34^,^35^. The other peak is the 2D mode at ~2687 cm^−1^, which is a two-phonon second-order Raman process. The integrated intensity ratio (*I*_2D_/*I*_G_) is 1.9, and the full width at half maximum (FWHM) of the 2D peak is 31 cm^−1^. These figures of merit first confirm the presence of as-transferred 1L graphene at the probed locations. At the exact same positions, two new peaks (defect-activated peaks) appear after PE-ALD HfO_2_ and PE-ALD Al_2_O_3._ Namely the D peak located at ~1356 cm^−1^ and the D’ peak (~1620 cm^−1^) at the right shoulder of the G mode. The D peak in sp^2^ graphene is activated by a double resonance Raman process in the presence of disorder and defects and is related to the breathing modes of carbon atoms in the vicinity of the K point in the Brillouin zone (see the work of Ferrari *et al.*^32^). The D peak can only be observed when the crystal symmetry is broken by point defects or at the edges of graphene^36^. The D’ mode corresponds to an independent defect-assisted intervalley process in graphene. It could be due to the presence of sp^3^ bonding. For as-transferred 1L graphene, the intensity of D peak is weak and ignored, as shown in the bottom spectrum. Since the size of the examined hexagon flake is large enough (larger than 15 μm from vertex to vertex) to make the measurement inside the crystalline region (in the center of hexagon) with a Raman laser spot diameter of about 1 μm, the boundary of the hexagons does not contribute to the spectrum here. After the dielectric depositions, the intensity of D peaks becomes very strong. This indicates that the dielectric depositions break the symmetry of the graphene lattice and induce structural defects in graphene. Moreover, the positions of the G peaks are slightly shifted, the FWHM of 2D peaks are broadened and the D’ peaks are separated from the G peaks. These characteristics indicate that graphene is disordered, but it is not completely etched and then still optically visible. We therefore use the area ratio *A*_D_/*A*_G_ between the integrated intensities of the D and G peaks to quantify the amount of disorder (*i.e.* the *A*_D_/*A*_G_ increases with increasing amount of disorder at low defect concentration range). The area ratio is preferred over the individual intensities since it accounts for variations in peak position, intensity and linewidth^37^.

**Figure 4:**
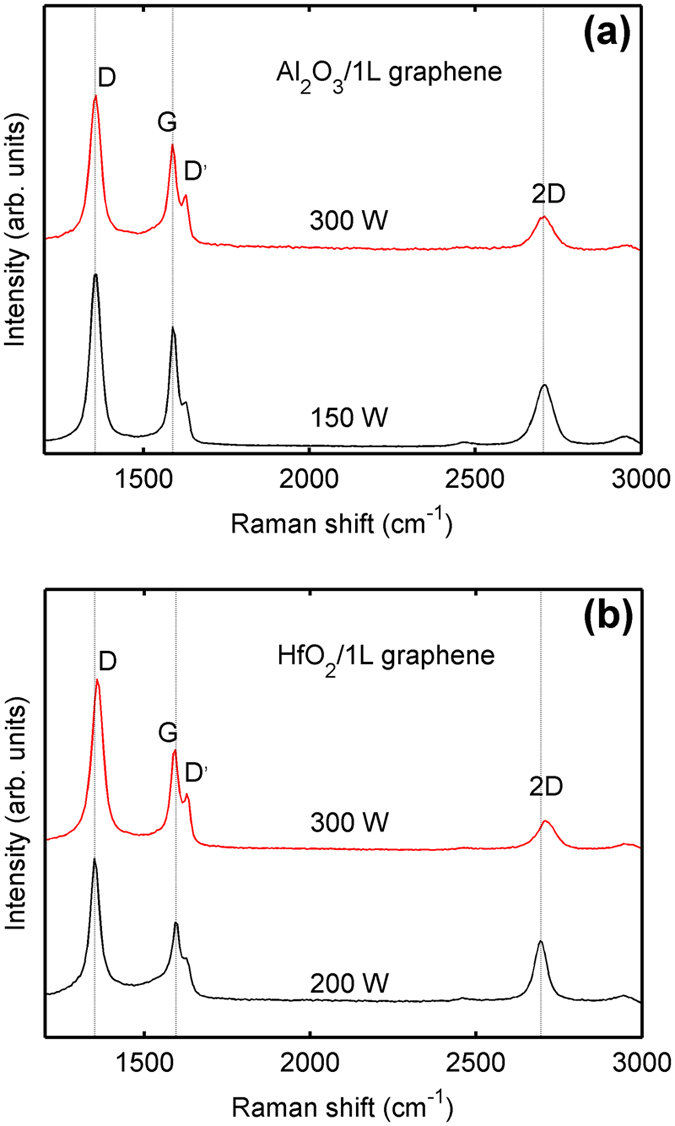
Raman spectra of monolayer graphene covered with (**a**) PE-ALD Al_2_O_3_ and (**b**) PE-ALD HfO_2_ for two different oxygen plasma power levels, respectively, (300 or 150 W) and (300 or 200 W).

To investigate the damage in graphene subjected to different oxygen plasma power levels, we reduce the oxygen plasma power from 300 to 200 W and 150 W for PE-ALD HfO_2_ and PE-ALD Al_2_O_3_, respectively. The corresponding Raman spectra of 1L graphene underlying the two dielectrics are shown in Fig. 4a,b, respectively. The D and D’ peaks exist even if the oxygen plasma power is reduced to 150 W. The *A*_D_/*A*_G_ ratios are very similar for the different power levels (300 and 200 W for HfO_2_, 300 and 150 W for Al_2_O_3_), implying that the amount of generated disorders in graphene is not correlated to the plasma power levels in this range of powers. For ALD Al_2_O_3_ on graphene, Lim *et al.* used nitrogen plasma to pretreat graphene^38^. They investigated the dependence of the number of defects on the nitrogen plasma power levels (30, 60, and 100 W). Their results show that the number of defects is increased with the nitrogen plasma power level. Our results suggest that for oxygen plasma powers above 150 W, the number of defects in 1L graphene has reached saturation.

**Figure 5:**
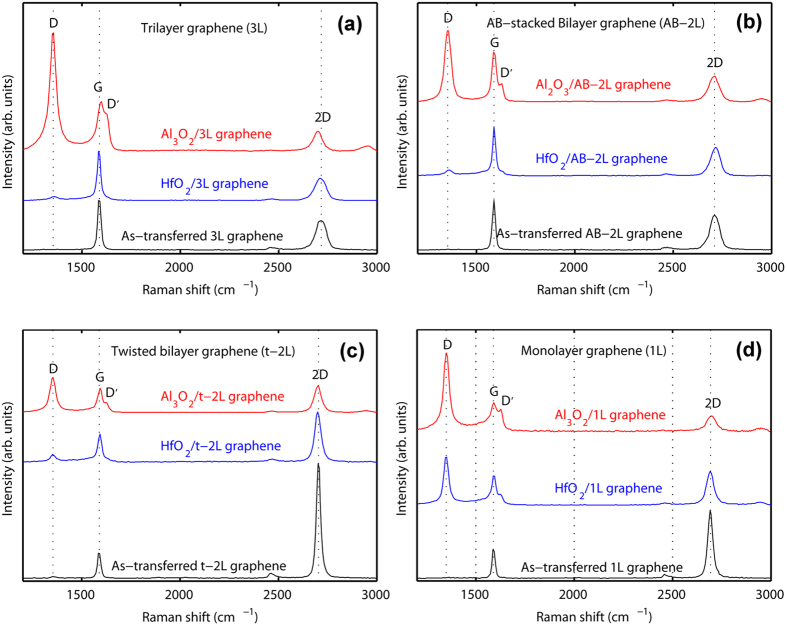
Raman spectra: (**a**) 3L graphene underlying HfO_2_ and Al_2_O_3_. (**b**) AB-2L graphene underlying HfO_2_ and Al_2_O_3_. (**c**) t-2L graphene underlying HfO_2_ and Al_2_O_3_. (**d**) 1L graphene underlying HfO_2_ and Al_2_O_3_.

The layer number is first identified by the color contrast of graphene under optical microscopy, followed by μRS measurements. Figure 5a–d shows the Raman spectra of 3L, AB-2L, t-2L and 1L graphene under HfO_2_ and Al_2_O_3_ with a thickness of about 5 nm, respectively. The Raman spectra of as-transferred graphene with various layer thicknesses (black curves) are also shown at the bottom of the figure as references. In the case of Al_2_O_3_/graphene (red curves), all the *A*_D_/*A*_G_ ratios are large and all the D’ peaks clearly separate from G peaks. These features indicate that all kinds of graphene are damaged during PE-ALD Al_2_O_3_. In the case of HfO_2_/3L graphene, the intensity of the D peak becomes very weak and the D’ peak even almost disappears (blue curve in Fig. 5a). The *A*_D_/*A*_G_ ratios of AB-2L and t-2L graphene are much smaller (blue curves in Fig. 5b,c). However, the spectrum of HfO_2_/1L graphene (blue curve in 5d) is similar to that of the Al_2_O_3_/graphene case. These results point out that in PE-ALD HfO_2_, FL graphene is less damaged than 1L graphene and the damage level of graphene decreases with the number of layers.

**Figure 6:**
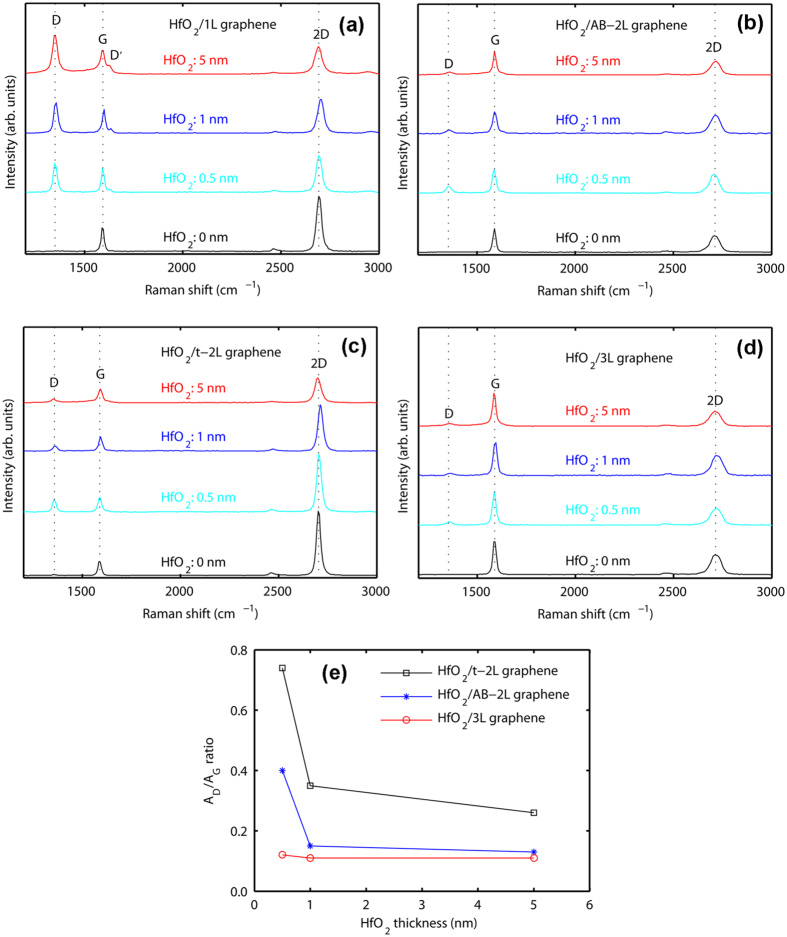
Raman spectra of HfO_2_/graphene for graphene layers with thicknesses of HfO_2_ (0, 0.5, 1 and 5 nm), (**a**) 1L, (**b**) AB-2L, (**c**) t-2L (t-2L), (**d**) 3L graphene and (**e**) all the *A*_D_/*A*_G_ ratios as a function of the HfO_2_ thickness.

We also investigate the influence of the HfO_2_ thickness on graphene. Figure 6a–d shows the Raman spectra for 1L, AB-2L, t-2L and 3L graphene under HfO_2_ with different thicknesses (0, 0.5, 1 and 5 nm), respectively. It can be seen from Fig. 6a that for different HfO_2_ thicknesses, the *A*_D_/*A*_G_ ratio of 1L graphene dramatically increases compared with that of as-transferred graphene. However, the changes in the Raman spectra of FL graphene are less drastic. Surprisingly, all the *A*_D_/*A*_G_ ratios decrease with increasing the thickness of HfO_2_ film (see Fig. 6e). The reason will be discussed later.

Finally, to identify if the HfO_2_ film or the hypothesized Hf-C exhibit some peaks in Raman spectra, μRS analysis is performed using high resolution (1800 gr/mm) gratings and three excitation laser energies in the visible range, namely 514.5 nm (2.41 eV), 488 nm (2.54 eV) and 633 nm (1.92 eV). We do not find any peak related to HfO_2_ or Hf-C in the measurement range from 0 to 4000 cm^−1^. This implies that the HfO_2_ film is amorphous due to the low growth temperature.

### X-ray photoelectron spectroscopy measurements

**Figure 7:**
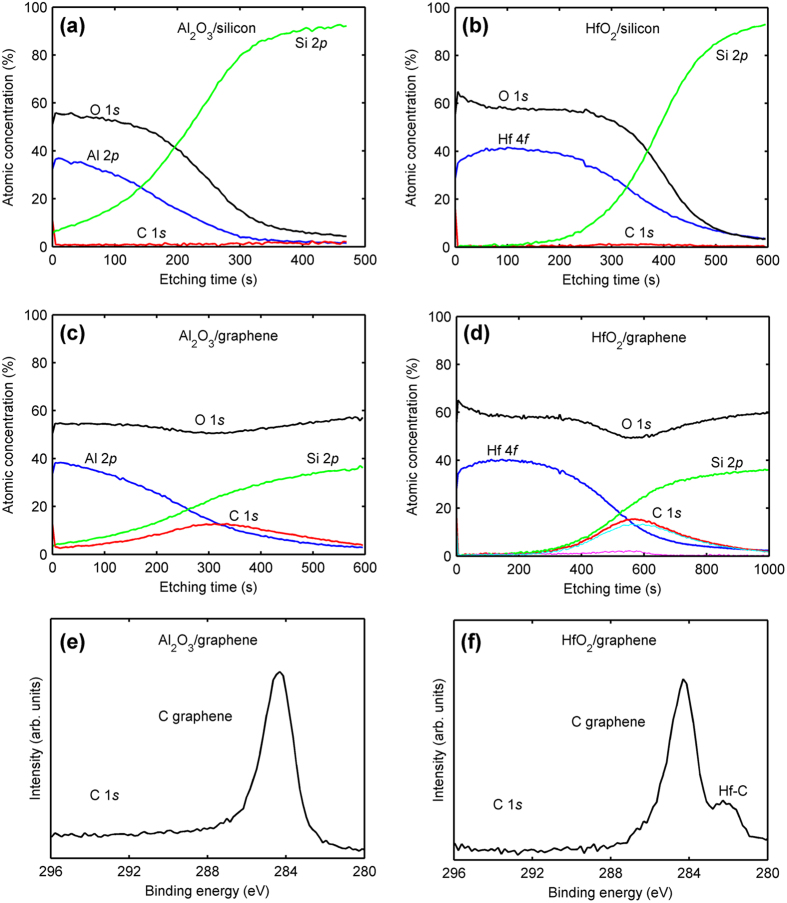
XPS depth profiles of (**a**) Al_2_O_3_/silicon, (**b**) HfO_2_/silicon, (**c**) Al_2_O_3_/graphene, and (**d**) HfO_2_/graphene samples. C 1*s* spectra of (**e**) Al_2_O_3_/graphene and (**f**) HfO_2_/graphene samples, corresponding to the maximum of the carbon profiles in (**c**) and (**d**), respectively.

We carry out *ex situ* XPS measurements to evaluate the impact of PE-ALD dielectrics on graphene. Four samples are sputtered with an Ar^+^ gun to perform a depth profile: 5.5-nm-thick Al_2_O_3_ and 5.9-nm-thick HfO_2_ are deposited either on silicon (as references) or on graphene/SiO_2_/silicon stacks (hereafter referred to as Al_2_O_3_/silicon, HfO_2_/silicon, Al_2_O_3_/graphene, and HfO_2_/graphene, respectively). Core level spectra are recorded from carbon (C 1s), oxygen (O 1s), hafnium (Hf 4f), aluminum (Al 2p), and silicon (Si 2p). The elemental composition of the dielectrics obtained from 20-nm-thick reference layers can be estimated from the ratios of the integrated intensities of the XPS spectra: [O/Hf] = 2.15 ± 0.1 and [O/Al] = 1.47 ± 0.05. These results testify to the good quality of the dielectrics. We now focus on the C 1s atomic concentration profile and spectra of each sample. Figure 7a,b illustrate the depth profiles of the Al_2_O_3_/silicon and HfO_2_/silicon samples, respectively. In both cases, a small amount of carbon (2% on average) is found in the profiles (except for a ~15% concentration corresponding to adventitious carbon on top of the dielectric layers). Moreover, a slight increase of the carbon concentration is observed when approaching the interface between the dielectric and silicon, most likely originating from residual contamination on silicon before PE-ALD. Figure 7c,d display the depth profiles of the Al_2_O_3_/graphene and HfO_2_/graphene samples, respectively. We can clearly identify the presence of graphene between the dielectric and the SiO_2_/silicon substrate. Figure 7e,f exhibit the C 1s spectra of the Al_2_O_3_/graphene and HfO_2_/graphene samples at the maximum of the carbon profiles, respectively. The main peak at 284.5 eV in both spectra corresponds to graphene. Strikingly, in contrast to the Al_2_O_3_/graphene sample, the HfO_2_/graphene sample displays an additional peak at 281.5 eV. This peak can be attributed to the formation of the metallic carbide Hf-C. Consequently, the HfO_2_/graphene profile in Fig. 7d can be fitted by its two components: C in graphene and C in Hf-C. At the interface, the Hf-C concentration reaches 2% of the total composition (see the cyan and magenta profiles in Fig. 7d, corresponding to C in graphene and C in Hf-C, respectively). However, it was reported by Engelhard *et al.*^39^ that Ar^+^ sputtering of ALD HfO_2_ induces the formation of Hf-C (at an ion-gun energy of 2000 eV), amounting to 1% of the total composition. To ascertain that the observed Hf-C peak is not related to sputtering (the Ar^+^ gun is operated on purpose at the very low energy of 200 eV in the hope of preventing Hf-C formation), we have performed an additional experiment. A 1L graphene is transferred onto an HfO_2_/SiO_2_/Si substrate and next subjected to sputtering in the same conditions as before. In the corresponding C 1 s spectrum, the carbide-related peak occurs as well, most likely due to intermixing between Hf and C during the erosion, resulting in Hf-C bonding. This means that, from the *ex situ* XPS analysis only, we cannot conclude if Hf-C forms during the PE-ALD process or during the erosion process or both. Nonetheless, as opposed to Al-C, this illustrates how easy it is to form Hf-C, since the Ar^+^ sputtering is operated at a very low energy of 200 eV. To further elucidate where Hf-C originates from requires an ALD apparatus fitted with an *in situ* XPS analyzer.

## Discussion

Although the optical image of the Al_2_O_3_/graphene/SiO_2_/Si (see Fig. 1 in the Supplementary Information) looks similar to that of the HfO_2_/graphene/SiO_2_/Si (see Fig. 2), all the graphene samples, regardless of their thickness, are significantly damaged in the PE-ALD Al_2_O_3_ process (see the Raman spectra of Fig. 5). This may be linked to the fact that the TMA-Al precursor itself does not react with graphene at temperatures lower than 400 °C^40^ 14. Al carbide is not formed during the initial TMA-Al precursor pulse, which is confirmed by the XPS data in Fig. 7e within the detection limits of the technique. This results in a delayed Al_2_O_3_ nucleation onto graphene. Only when graphene is subjected to the first or few oxidant precursor pulses (which is analogous to a pretreatment by oxygen plasma), the Al_2_O_3_ film starts to nucleate and then grow. Unfortunately, 1L graphene, and even FL graphene, have been degraded during the pretreatment. On the contrary, the XPS results have demonstrated that the Hf-C bonds are quite easily formed, whatever the origin of the bonding. We hypothesize that, during the initial TDMA-Hf precursor pulse, a chemical reaction occurs between the Hf atoms and the C atoms constituting graphene to form Hf-C (its formation temperature is 25 °C)^39^. Hf in Hf-C bonds may act as a uniform and active template for the subsequent HfO_2_ growth. Subsequently, the coverage of the first HfO_2_ layer protects the underlying graphene layer from damage during the following deposition cycles. This may also explain why HfO_2_ rather than Al_2_O_3_ can be directly grown on graphene without out-of-plane covalent functional groups, in low-temperature thermal ALD process^41^,^42^.

On the other hand, the origin of the inverse dependence of the *A*_D_/*A*_G_ ratio on the number of graphene layers is not clear. It may be due to the interlayer interactions between the HfO_2_ layer and graphene or a direct consequence of the HfO_2_ deposition process or both. Previous works about the impact of the graphene thickness on its physical properties suggest that graphene’s rigidity may increase with increasing number of layers^43^. More precisely, when 1L graphene is deposited on top of the SiO_2_ substrate, it conforms more easily to the surface morphology of the underlying substrate, thereby more prone to deform compared with FL graphene. Oxygen plasma modification of 2L and FL graphene has been studied in the literature. Calculation results^44^ predict that oxidized 2L graphene, unlike 1L, still retains its intrinsic properties even if the oxygen density is as high as 50%. Electrical experimental results in the work of Felten *et al.*^45^ show that only the top layer of 2L graphene is chemically modified, while the bottom layer maintains its structural integrity. Moreover, it was reported that the chemical modification of FL graphene occurs layer by layer^46^,^47^. The Raman spectra of HfO_2_/graphene (blue curves in Fig. 5) show that the *A*_D_/*A*_G_ ratio of 1L graphene is much larger than that of FL graphene and the D’ peak is separated from the G peak in the Raman spectrum of 1L graphene. The formation of Hf-C bonds means a chemical adsorption of Hf atoms on graphene and the conversion of the bond from sp^2^ to sp^3^, with a resulting increase of the D peak and separation of the D’ peak. In addition, the oxygen plasma pulse is very aggressive toward 1L graphene^48^. Besides, the poor rigidity of 1 L graphene results in its fracture and wrinkling^49^. All these effects would induce damages in the graphene lattice *e.g.* vacancies, dislocations and dangling bonds. In sharp contrast to 1L, the *A*_D_/*A*_G_ ratios of FL graphene significantly decrease. Hf-C bonds are only present on the top layer due to preferential chemisorption of Hf atoms. Since the plasma pulse is less reactive to FL graphene than 1L^50^ and graphene becomes more rigid with the number of layers, this may lead to less damage in FL graphene.

The *I*_2D_/*I*_G_ ratio and the FWHM of the 2D peak can be used to easily distinguish 1L and t-2L graphene. More specifically, pristine 1L graphene has a *I*_2D_/*I*_G_ ratio of about 2 and a FWHM of 35 cm^−1^, while the *I*_2D_/*I*_G_ ratio and the FWHM of t-2L graphene are about 6 and 28 cm^−1^, respectively^51^. Our results reveal another interesting behavior: after depositing a 5-nm-thick HfO_2_ film, t-2L graphene presents “1L-like” features. In other words, when the thickness of the HfO_2_ film increases from 0 to 5 nm, the *I*_2D_/*I*_G_ ratio reduces from 5.83 to 1.97 and the 2D peak FWHM increases from 26 to 38 cm^−1^ (see Fig. 6c). It is emphasized here that the *I*_2D_/*I*_G_ ratio of about 2 and the 2D peak FWHM of about 35 cm^−1^ are the features of 1L graphene^32^, while t-2L graphene has a *I*_2D_/*I*_G_ ratio of about 6 and a 2D peak FWHM of about 28 cm^−1^ (see ref. 51). We suggest that the C atoms in the top layer of t-2L intermixing with Hf atoms and O atoms form an interfacial layer, which is a complex amorphous layer. It is supported by the of the cross-sectional HAADF-STEM image as shown in the insert of Fig. 2c. This is consistent with a previous work^52^. In Fig. 6c, we can see that the spectrum of t-2L gradually becomes 1L-like upon augmenting the HfO_2_ thickness. We make the assumption that, below 5 nm, the interfacial layer is not completely formed. In contrast, for 5 nm, the top graphene layer is entirely incorporated into the interfacial layer, leaving a stack made up of HfO_2_/amorphous interlayer/1L-like graphene. μRS cannot detect that interfacial layer because it is amorphous. On the other hand, Felten *et al.*^45^ observed a similar behavior with AB-2L under different plasma conditions. Indeed, they show by electrical measurements that after long-time and mild plasma treatment, 1L becomes an insulator, while AB-2L graphene still retains its ambipolar property with a relatively high charge mobility. They attribute these facts to the chemically modified top graphene layer and decoupling between the top and bottom layer. However, we did not observe the same behavior for AB-2L and 3L.

As shown in Fig. 6e, the *A*_D_/*A*_G_ ratios of FL graphene decrease with increasing the thickness of HfO_2_. This is also consistent with the above discussions. More specifically, the increase of the HfO_2_/graphene thickness makes it more difficult to conform to the surface morphology of the underlying substrate, and as a consequence graphene is less prone to deform.

It is worth emphasizing that FL graphene may be a prospective material with regard to applications such as transistors and sensors. For instance, it has been reported that the sheet resistance of 2L graphene is smaller than that of 1L graphene and the low-frequency 1/f noise in the transistor (30-nm gate length) made from 2L graphene is strongly suppressed compared with 1L graphene transistors^53^,^54^.

## Conclusion

We have investigated the structural damage in graphene underlying dielectrics (HfO_2_ and Al_2_O_3_) deposited by remote PE-ALD. Our results show that FL graphene is less damaged than 1L graphene; the damage level of FL graphene decreases not only with the number of graphene layers but also with the thickness of HfO_2_. Interestingly, the Raman spectrum of t-2L graphene underlying HfO_2_ presents the features of that of as-transferred 1L graphene. XPS measurements indicate that Hf-C is easily formed. After coverage by the first HfO_2_, the bottom graphene layer has an additional protection. The oxygen plasma pulse in PE-ALD is less reactive to FL graphene than 1L. Graphene rigidity increases with the number of graphene layers. Moreover, it also increases with the thickness of HfO_2_. These may be the reasons of FL graphene less damaged in PE-ALD HfO_2_. Therefore, FL graphene, more particularly, t-2L graphene allows for controlling/limiting the defect formation during the PE-ALD HfO_2_ process and might be a prospective material for applications such as graphene-based transistors and sensing devices. It appears that the thickness of PE-ALD HfO_2_ can be arbitrarily scaled down to 5 nm. Our results open up direct perspectives for FL graphene and HfO_2_ gate dielectric in graphene-based transistor applications.

## Methods

### APCVD graphene conditions

Graphene is synthesized by APCVD with dilute methane (5% in argon) as hydrocarbon precursor on copper foils (Alfa Aesar #13382). The samples are grown at 1000 °C for 1 h under flows of 500 sccm of argon, 20 sccm of hydrogen, and 0.2 sccm of dilute methane. Graphene is then transferred onto 300-nm-thick SiO_2_/Si substrates (to easily observe graphene with a conventional white light microscope) by the usual method based on PMMA, after etching the copper foil in ammonium persulfate.

### Process conditions for PE-ALD of dielectrics on graphene

HfO_2_ and Al_2_O_3_ films are deposited on graphene/SiO_2_/Si stacks, by PE-ALD (Fiji F200 from Ultratech/Cambridge NanoTech Inc., MA) at 250 °C. The plasma source, inductively coupled at 13.56 MHz, is far away from the samples. It is very important to note that the distance between the plasma source and sample location is larger than 40 cm since the type and concentration of the reactive species (electrons, ions, and radicals) strongly depend on this distance. Outside of the glow discharge, only long lifetime radicals are present while ions and electrons recombine quickly.

In order to remove the native stress and polymethyl methacrylate (PMMA) residues from growth and transfer, the graphene samples on the SiO2/Si substrate first are annealed at 250 °C for 2 h in the deposition chamber under pressure of 80 mtorr (argon gas). During both dielectric film depositions, the pulse duration of the oxygen plasma (oxidant precursor) is 10 s for each cycle. The flows of the oxygen plasma and the argon carrier gas are 20 and 200 sccm, respectively. The metallic and oxidant precursor pulses are separated by a short argon purge of 5 s. To obtain uniform dielectric films and avoid graphene etching, the metallic precursors are first pulsed on the graphene surface. The metallic precursors can adsorb or react with carbon to form the related metal oxides following the first oxidant precursor pulse. In contrast, if the first pulse is a single cycle of oxygen plasma, the graphene surface is possibly damaged or non-uniform dielectric films are formed. The other parameters related to the metallic precursors and the final thickness of both dielectric films are listed in Table 1. The thickness of the dielectric films is measured by *in situ* ellipsometry from reference films directly deposited on silicon substrates. The composition of the dielectric films is characterized by XPS. In order to investigate the damage level of graphene upon different oxygen plasma power levels, HfO2 and Al2O3 films are deposited on graphene/SiO2/Si stacks with nominal 300 W and with reduced oxygen plasma power of 200 and 150 W, respectively.

### Raman spectroscopy system

A LabRam HR 800 confocal laser system from Horiba Jobin Yvon was used for the acquisition of the Raman spectra. The measurements are performed at room temperature with a laser wavelength λ = 514 nm in backscattering geometry. The laser beam is focused on the center of hexagons and a 100 × objective (NA = 0.95) is used to collect the signal. The incident power is kept below 1 mW. Low resolution (150 g/mm) and high resolution (1800 g/mm) gratings are used for the measurements.

### XPS characterization

A ThermoFisher Scientific K-alpha spectrometer is employed. It is equipped with a monochromatized Al *K*α1,2 x-ray source and a hemispherical deflector analyzer. The spectra are recorded at constant pass energy (150 eV for depth profiling and survey; 30 eV for high resolution spectra). A flood gun (low energy electrons and Ar ions) is used during all the measurements. During the sputtering, the Ar^+^ ion gun is operated at a low energy (200 eV), with an erosion time of 5 s per cycle, and the analysis is done in snapshot mode. The XPS data are treated with the Avantage software. High resolution spectra are fitted by Gaussian-Lorentzian lineshapes with an Avantage “smart” background (*i.e.* a Shirley background in most cases, or a linear background in case the lineshape decreases with increasing BE).

## Additional Information

**How to cite this article**: Tang, X. *et al.* Damage evaluation in graphene underlying atomic layer deposition dielectrics. *Sci. Rep.* **5**, 13523; doi: 10.1038/srep13523 (2015).

## Supplementary Material

Supplementary Information

## Figures and Tables

**Table 1:** Process conditions in PE-ALD and thicknesses of the two dielectric films.

Dielectric Name	HfO_2_	Al_2_O_3_
Precursor acronym	TDMA-Hf	TMA
Precursor chemical formula	[(CH_3_)_2_N]_4_Hf	Al_2_(CH_3_)_6_
Precursor temperature (°C)	75	25
Precursor pulse duration (s)	0.25	0.06
Cycle number (cycle)	55	55
Final thickness (nm)	5.9	5.5
