# Supplementary material for: Damage evaluation in graphene underlying atomic layer deposition dielectrics
Source: Sci Rep. 2015 Aug 27;5:13523. doi: 10.1038/srep13523 (PMC4550929; doi:10.1038/srep13523)
Supplement: Supplementary Information [file srep13523-s1.doc]

**Damage evaluation in graphene underlying atomic layer deposition dielectrics**

(Supplementary Information)

*Xiaohui Tang^1,*^, Nicolas Reckinger^2,3^, Olivier Poncelet^1^, Pierre Louette^3^, Ferran Ureña^1^, Hosni Idrissi ^5,6^, Stuart Turner^5^, Damien Cabosart^4^, Jean-François Colomer^2,3^, Jean-Pierre Raskin^1^, Benoit Hackens^4^, and Laurent A. Francis^1^*

Figure 1. Optical images: (a) as-transferred graphene on SiO_2_/Si substrate, (b) Al_2_O_3_/graphene/SiO_2_/Si stack (Al_2_O_3_ film has a thickness of 5 nm).
